# MTA2 silencing attenuates the metastatic potential of cervical cancer cells by inhibiting AP1-mediated MMP12 expression via the ASK1/MEK3/p38/YB1 axis

**DOI:** 10.1038/s41419-021-03729-1

**Published:** 2021-05-06

**Authors:** Chia-Liang Lin, Tsung-Ho Ying, Shun-Fa Yang, Hui-Ling Chiou, Yong-Syuan Chen, Shao-Hsuan Kao, Yi-Hsien Hsieh

**Affiliations:** 1 Institute of Medicine, Chung Shan Medical University, Taichung, Taiwan; 2 Department of Obstetrics and Gynecology, School of Medicine, College of Medicine, Chung Shan Medical University, Taichung, Taiwan; 3 Department of Obstetrics and Gynecology, Chung Shan Medical University Hospital, Taichung, Taiwan; 4 Department of Medical Research, Chung Shan Medical University Hospital, Taichung, Taiwan; 5 School of Medical Laboratory and Biotechnology, Chung Shan Medical University, Taichung, Taiwan

**Keywords:** Oncogenes, Cell invasion

## Abstract

Metastasis-associated protein 2 (MTA2) is a transcription factor that is highly associated with matrix metalloproteinase 12 (MMP12). Thus, we hypothesized that MTA2 may regulate MMP12 expression and is involved in cervical cancer metastasis. Results showed that MTA2 and MMP12 were highly expressed in cervical cancer cells, and MTA2 knockdown reduced MMP12 expression and inhibited the metastasis of cervical cancer cells in xenograft mice. MMP12 knockdown did not influence the viability of cervical cancer cells but clearly inhibited cell migration and invasion both in vitro and in vivo. MMP12 was highly expressed in cervical tumor tissues and correlated with the poor survival rate of patients with cervical cancer. Further investigations revealed that p38 mitogen-activated protein kinase (p38), mitogen-activated protein kinase kinase 3 (MEK3), and apoptosis signal-regulating kinase 1 (ASK1) were involved in MMP12 downregulation in response to MTA2 knockdown. Results also demonstrated that p38-mediated Y-box binding protein1 (YB1) phosphorylation disrupted the binding of AP1 (c-Fos/c-Jun) to the MMP12 promoter, thereby inhibiting MMP12 expression and the metastatic potential of cervical cancer cells. Collectively, targeting both MTA2 and MMP12 may be a promising strategy for the treatment of cervical cancer.

## Introduction

Cervical cancer is one of the most life-threatening malignancy in women in the developing countries^1,2^. Various types of treatments combining surgeries, radiotherapy, and chemotherapy have been developed to heal precancerous lesions and eradicate aggressive and malignant cervical carcinomas^3^. However, many patients with cervical cancer still suffer from recurrence and distal metastasis of cancer after treatments. Therefore, the molecular mechanism of cervical carcinogenesis must be comprehensively explored to develop more specific and effective treatments to this disease.

Distal metastasis is often found in tumor recurrence, and it is the major cause of death for most patients with cancer. Compared with the mechanisms for tumor initiation and regulation of tumor growth, the molecular process of metastasis remains unclear. Many dynamic cellular processes are involved in the dissemination of cancer cells from the primary site to distant sites, including changes in cytoskeletal structure, cell–cell and cell–extracellular matrix (ECM) interaction, and ECM degradation^4^. Matrix metalloproteinases (MMPs) belong to the zinc protease family and play a pivotal role in tumor metastasis mainly by inducing ECM degradation and stimulating tumor growth^5^.

Metastasis-associated protein 2 (MTA2) is a transcriptional regulator belonging to the metastasis tumor-associated family. Previous studies demonstrated that MTA2 plays an important role in cytoskeletal organization and transcription, as well as in the promotion of the metastatic potential of tumor cells^6,7^. However, the role of MTA2 in the metastatic ability of cervical cancer cells remains poorly understood. Here, we showed that MTA2 is highly expressed in cervical tumor tissues and is associated with the poor prognosis of cervical cancer. To explore the role of MTA2 in the metastatic potential of cervical cancer cells, we established MTA2-knockdown cervical cancer cells to investigate the effects of MTA2 knockdown on cell motility, invasiveness, and in vivo metastasis.

## Materials and methods

### Reagents and antibodies

Chemicals and antibodies without specific indication were purchased from Sigma-Aldrich (St. Louis, MO, USA). The antibodies source and dilution factor: MTA2 (sc-55566; 1:1000), ERK (sc-514302; 1:1000), p-p38 mitogen activated protein kinase (p-p38) (sc-166182; 1:1000), p38 (sc-7972; 1:1000), p-JNK (sc-6254; 1:1000), JNK (sc-571; 1:1000), ASK1 (sc-5294; 1:1000), p-MEK3 (sc-8407; 1:1000), MEK3 (sc-960; 1:1000), c-Jun (sc-74543; 1:1000), c-Fos (sc-8047; 1:1000), lamin B (sc-374015; 1:2000), YB1 (sc-18057; 1:1000), β-actin (sc-69879; 1:2000), and peroxidase-conjugated antibodies against mouse IgG or rabbit IgG (1:10000) were obtained from Santa Cruz Biotechnology (Santa Cruz, CA, USA). MMP12 (ab66157; 1:1000) were purchased from Abcam (Cambridge, UK). Phospho(p)-ASK1 (#3764; 1:1000), phospho(p)-ERK1/2 (#9101; 1:1000) and phospho(p)-YB1 (#2900; 1:1000) were purchase from Cell Signaling Technology (Beverly, MA, USA). Human cervix cancer array (CR805) were purchased from US Biomax, Inc (Rockville, MD, USA).

### Immunohistochemical staining

Tissues were fixed, blocked, sliced, and incubated with primary antibodies against human MTA2 or anti-MMP12. After washing with PBS, the reacted sections were incubated with peroxidase-conjugated second antibodies and then reacted with diaminobenzidine solution for signal visualization as previously described^8^. Protein expression in the immunohistochemical staining was quantified according to the immunoreactivity score.

### Reverse transcription and real-time quantitative polymerase chain reaction

Cells were harvested, and total RNA was extracted using Isol-RNA-Lysis Reagent (Gaithersburg, MD, USA). Complementary DNA was acquired via reverse-transcription of total RNA by using the ReverTra Ace qPCR RT Master Mix kit (Toyobo, Japan). Reverse transcription polymerase chain reaction (RT-PCR) was conducted using GoScript Reverse Transcriptase (Madison, WI, USA). Quantitative RT-PCR (qRT-PCR) was performed using the StepOne Real-Time PCR System (Applied Biosystems, Foster City, CA, USA). The primers used for qRT-PCR were: MTA2, (F) 5ʹ-TGT ACC GGG TGG GAG ATT AC-3ʹ, (R) 5ʹ- TGA GGC TAC TAG AAA TGT CCC TG-3ʹ; MMP12, (F) 5ʹ- CAT GAA CCG TGA GGA TGT TGA -3ʹ, (R) 5ʹ-GCA TGG GCT AGG ATT CCA CC-3ʹ; and glyceraldehyde 3-phosphate dehydrogenase (GAPDH), (F) 5ʹ-CAT CAT CCC TGC CTC TAC TG-3ʹ, (R) 5ʹ-GCC TGC TTC ACC ACC TTC-3ʹ (Mission Biotech, Taipei, Taiwan). Relative quantitation of gene expression was normalized with endogenous GAPDH via the 2^-ΔΔCt^ method.

### Cell culture and gene knockdown by shRNA lentivirus infection

Human cervical cancer cell lines HeLa, SiHa, and C33A were cultured in Dulbecco’s modified Eagle’s medium (DMEM) containing 10% v/v fetal bovine serum (FBS, HyClone, Thermo Fisher, Waltham, MA, USA), 50 U/mL penicillin, and 50 μg/mL streptomycin (Sigma-Aldrich) in a humidified incubator supplied with 5% CO_2_. MTA2 and MMP12 knockdown was achieved by the transfection of specific shRNA lentiviral constructs (shMTA2 and shMMP12, National RNA Interference Core Facility, Institute of Molecular Biology, Academia Sinica, Taipei, Taiwan). The target sequences of shMTA2 and shMMP12 were 5ʹ-AGGGAGTGAGGAGTGAATTAA-3ʹ and 5ʹ-CTTGCTTGACTCTACTATTAA-3ʹ, respectively. Luciferase lentiviral construct (shLuc) was used as control. The transfection assay was conducted according to the guidelines of the Institutional Biosafety Committee of Chung Shan University. HEK293T cells were used for target lentivirus production. Both HeLa and SiHa cells with stable MTA2- and MMP12-knockdown were generated by lentiviral infection and then followed by puromycin selection for 2 weeks. Inhibition efficiency of the shRNA constructs was confirmed via Western blot and qRT-PCR.

### Western blot analysis

Cells were washed with PBS, collected, and then lysed with RIPA buffer (Cell Signaling, Beverly, MA, USA) for protein extraction. The extracted proteins were separated by a 10% SDS–polyacrylamide gel and then transferred onto a PVDF membrane (Immobilon P, Merck Millipore, Billerica, MA, USA). The membrane was blocked with 2% skimmed milk and then incubated with primary antibodies, peroxidase-conjugated secondary antibody (Santa Cruz Technology), and with a chemiluminescence substrate (GE Healthcare, London, UK) as previously described^9^. Chemiluminescent signals were acquired and semi-quantitated by a Luminescent Image Analyzer LAS-4000 mini (GE Healthcare).

### Assessment of lung metastasis using xenograft model

Female BALB/c-nude mice aged 5 weeks were purchased from National Laboratory Animal Center (Taipei, Taiwan) and handled according to the approval of the Animal Ethics Committee of Chung Shan Medical University. The mice were kept in cages with a regular 12 h light/dark cycle and provided access to a standard rodent diet ad libitum. The HeLa or SiHa cells infected with shLuc, shMTA2, or shMMP12 lentivirus (1 × 10^6^) were then subcutaneously injected into the mice (*n* = 5 for each group). After 6-week maintenance, the mice were sacrificed in a compressed CO_2_ chamber. The number of lung tumor nodules was counted, and the weight of lungs was recorded. The lung tumor tissues were subjected to immunohistochemical staining by using specific antibodies.

### Cell viability assay

Cell viability was assessed via MTT assay as previously described^9^. The viable cells were proportional to the absorbance, and the cell viability was presented as a percentage of control.

### Migration and invasion assay

Cell migratory and invasive potentials were assessed by migration and invasion assays, respectively, by using 24-well modified Boyden chambers containing membrane filter inserts with 8 μm-wide pores (Corning Incorporated Life Sciences, Tewksbury, MA, USA). For invasion assay, membrane filter inserts were precoated with Matrigel. The lower compartment was filled with 20% FBS/DMEM. Cells were placed in the upper part of a Boyden chamber and incubated for 16 h. The numbers of transmigrated cells on the lower side of the filter were then stained with crystal violet and counted. Average cell numbers were acquired from five randomly selected fields.

### Public database analysis and assessment

The Gene Expression Profiling Interactive Analysis (GEPIA) database (http://gepia.cancer-pku.cn/), an online database containing RNA expression information and survival data from the TCGA and the GTEx projects, was used for the analysis and assessment of expression of MTA2 and MMP12 and their correlation to the survival rate of patients with cervical cancer^10^. The PROMO database (http://alggen.lsi.upc.es) was used to identify the putative transcription factor binding sites of MMP12 promoter.

### Gene silencing of ASK1, YB1, MEK3, and p38

Small inhibitory RNAs (siRNAs) specifically targeting ASK1 (siASK1; a pool of sc-29748A, sc-29748B, and sc-29748C), MEK3 (si-MEK3; sc-43924), YB1 (si-YB1; sc-38634), and p38 (si-p38; a pool of sc-29433A, sc-29433B, sc-29433C, and sc-29433D), as well as a scrambled control siRNA, were constructed by and obtained from Santa Cruz Biotechnology (Santa Cruz, CA, USA). The siRNAs were transfected using Lipofectamine RNAiMAX Transfection Reagent (Thermo Fisher Scientific, Waltham, MA, USA) following the manufacturer’s protocol.

### Immunofluorescence staining

Cells were fixed using 4% formaldehyde, incubated with the indicated primary antibodies, and then incubated with Alexa Fluor-labelled secondary antibody (Jackson ImmunoResearch Laboratories, West Grove, PA, USA). DAPI staining was used for the detection of cell nucleus. Fluorescence images were obtained using a laser scanning confocal microscope system (Zeiss 510 meta, Zeiss, Oberkochen, Germany).

### Luciferase reporter assay

Stable MTA2-silencing SiHa cells were transfected with human MMP12-promoter-luciferase and β-gal vector to evaluate transfection efficiency. The activity assay for MMP12-promoter luciferase and β-gal was performed in accordance with the manufacturer’s instruction (Luciferase Assay Kit, Promega, Madison, WI, USA). The primers used for luciferase reporter were listed below. MMP-12 promoter (−2000/+1) sequences: Forward, 5ʹ-CTCGAGCAGTATTAGAAGAAACTTCAT-3ʹ, Reverse, 5ʹ-AGATCTTAGTTTGATTATTTTCCT-3ʹ; MMP-12 promoter (−1000/+1) sequences: Forward, 5ʹ-CTCGAGCAGTATTAGAAGAAACTTCAT-3ʹ, Reverse, 5ʹ- AGATCTGCACCCTACCGCACC-3ʹ.

### Chromatin immunoprecipitation

Chromatin immunoprecipitation was conducted as previously described^11^. DNA was immunoprecipitated by antibodies against c-Fos or c-Jun, purified using QIAquick PCR Purification Kit (Qiagen), and then analyzed via PCR by using specific primers for MMP12 promoter regions. The primers used for ChIP were listed below. MMP-12 promoter (−122/−115) sequences: Forward, TTGATCCATTGTCGTCTGAA, Reverse, 5ʹ-TGTAAACTTCTAAACGGATC-3ʹ; MMP-12 promoter (−1801/−1793) sequences: Forward, 5ʹ-CAAACCTCAGCTATGCCACC-3ʹ, Reverse, 5ʹ-GGAATAGTAATAAATGTTGA -3ʹ.

### Statistical analysis

Quantitative data were presented as means ± standard errors from three independent experiments. ANOVA and an unpaired two-tailed Student’s *t* test were used to determine the significance of differences. Overall survival curve of the patients with cervical cancer was calculated via the Kaplan–Meier method. The relationship between MTA2 and MMP12 was assessed by Spearman rank correlation. *P* < 0.05 was considered as statistically significant.

## Results

### MTA2 mediates MMP12 expression and is highly expressed in human cervical cancer cells, and is associated with the lung metastasis of cervical cancer

**Fig. 1:**
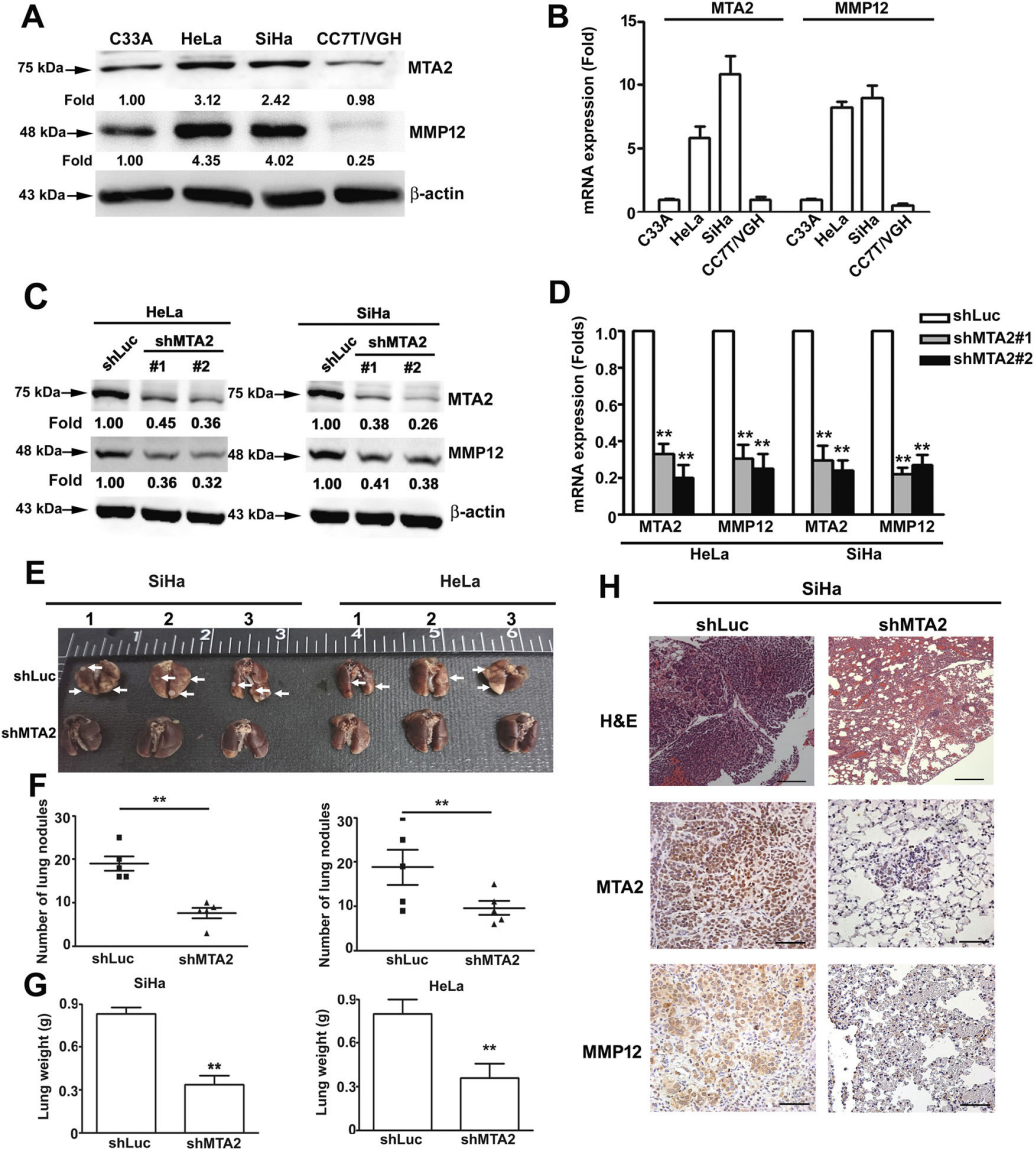
MTA2 expression in human cervical cancer cells and its correlation with MMP12 and cervical cancer lung metastasis. **(A)** Protein level of MTA2 and MMP12 in human cervical cancer cells as determined by Western blot. (**B)** mRNA expression of MTA2 and MMP12 in human cervical cancer cells as determined by qRT-PCR. (**C)** Protein level of MTA2 and MMP12 in MTA2-knockdown HeLa and SiHa cells as determined by Western blot. (**D)** mRNA expression of MTA2 and MMP12 in MTA2-knockdown HeLa and SiHa cells as determined by qRT-PCR. (**E**–**H)** MTA2-knockdown cells were subjected to in vivo xenograft analysis for assessment of (**E**) lung metastasis, (**F**) numbers of lung nodules, (**G**) weights of lungs, and (**H**) histological change in lung tissues as indicated by IHC staining. **P < 0.01 compared with the shLuc cells or shLuc-group.

By using human proteinase array analysis, we previously found that MTA2 knockdown decreased the MMP12 production^11^. Thus, we first investigated whether MTA2 regulates MMP12 in human cervical cancer cell lines. As shown in Fig. 1A, B, MTA2 and MMP12 were highly expressed in cervical cancer HeLa and SiHa cells but weakly expressed in CC7T/VGH and C33A cells. We then silenced MTA2 expression by using specific shRNA. MTA2-knockdown not only clearly inhibited MTA2 expression but also substantially reduced MMP12 expression in HeLa and SiHa cells (Fig. 1C, D). Furthermore, MTA2 overexpression resulted in increased MTA2 and MMP12 protein levels and enhanced migration and invasion of C33A and CCT7/VGH cells (Supplemental Fig. S1). We further explored the in vivo effects of MTA2-knockdown on the metastasis of cervical cancer cells by using a xenograft model. Results showed that the lung metastasis and lung weight of MTA2-knockdown SiHa and HeLa cells were considerably diminished compared with that of the shLuc group (*P* < 0.01, Fig. 1E–G). In addition, MTA2 and MMP12 protein expression levels were clearly decreased in the metastasized tumors derived from MTA2-knockdown SiHa cells (Fig. 1H).

### MMP12 expression is associated with the metastatic competence of cervical cancer cells both in vitro and in vivo and linked to poor survival of patients with cervical cancer

**Fig. 2:**
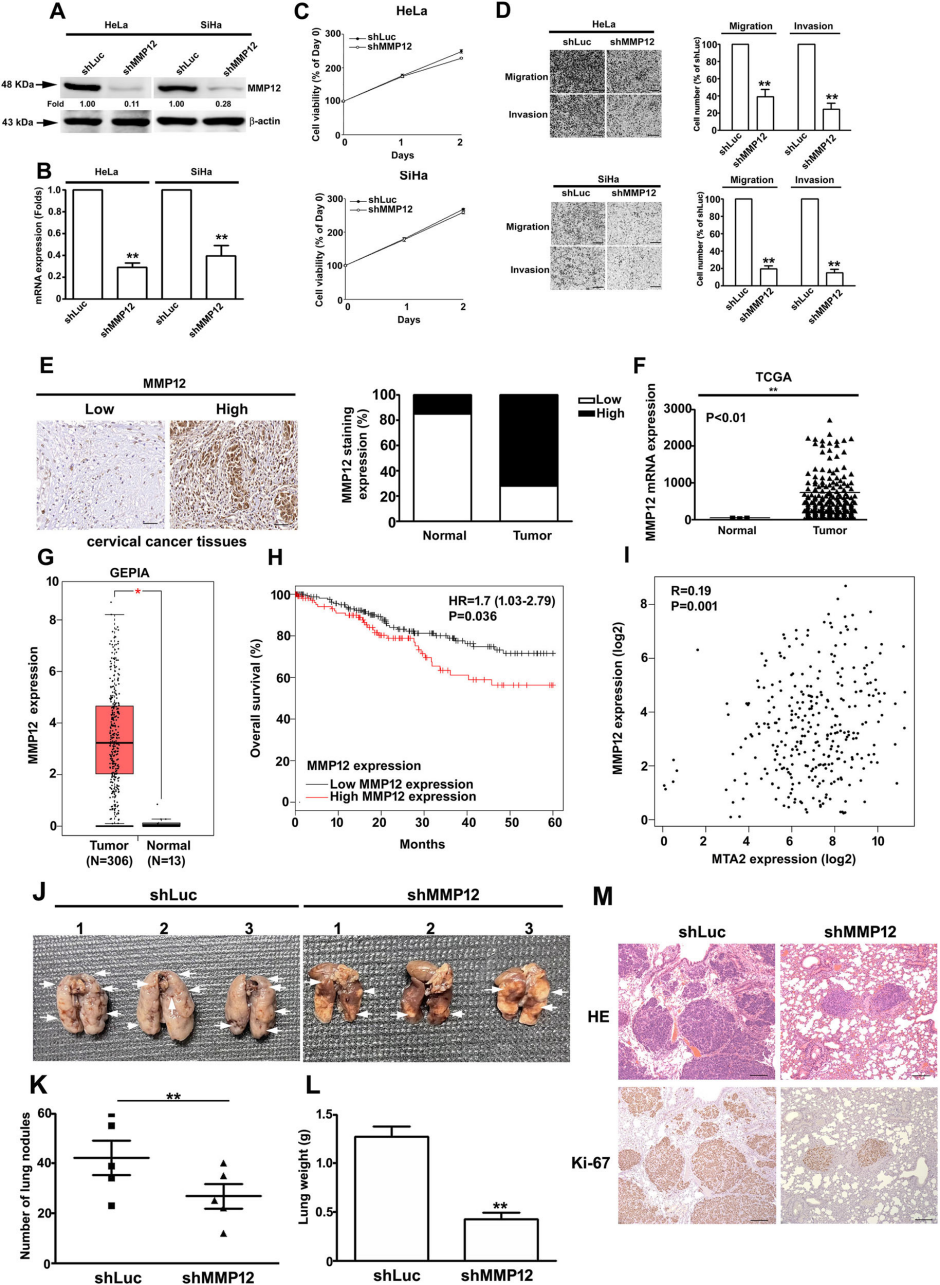
MMP12 knockdown reduced the cell migration and invasion of human cervical cancer cells both in vitro and in vivo, and MMP12 expression in cervical tumors and its correlation with patient survival rates and MTA2 expression. Cells were transfected with shRNAs against MMP12 (shMMP12). The protein and mRNA expression of MMP12 (**A**, **B**), cell viability (**C**), and cell migration and invasion (**D**) were then assessed by Western blot, RT-PCR, MTT assay, transmigration assay, and invasion assay, respectively. (**E)** Protein expression of MMP12 in cervical cancer tissues was demonstrated using IHC staining. (**F)** mRNA expression of MMP12 in normal and tumor tissues in cervical cancer as determined by using the TGCA database. **G** MMP12 expression between tumor and normal tissues in cervical cancer as determined by using GEPIA. **H** Kaplan–Meier survival analysis for low and high MMP12 expression in cervical tumor tissues from the TCGA database. (**I)** Correlation between MMP12 and MTA2 expression in cervical tumor tissues from the TCGA database. Cells transfected with shLuc was used as sham control. (**J**–**M)** Lung metastasis of shLuc- and shMMP12-SiHa cells in xenograft mice. (**J)** Phenotype of lung nodules, (**K**) number of lung nodules, (**L**) lung weights, and (**M**) histological examination of tumor nodules via HE staining and Ki-67 expression by mmunohistochemistry assay. Quantitative results in (**B**–**D**) were obtained from three independent analyses. *P < 0.05, compared with normal tissues. **P < 0.01 compared with shLuc cells or normal tissues.

We then explored the role of MMP12 in the carcinogenesis of cervical cancer. We definitively silenced MMP12 expression in HeLa and SiHa cells by using specific shRNAs (Fig. 2A, B, *P* < 0.01 compared with control). MMP12-knockdown did not influence cell viability (Fig. 2C) but remarkably attenuated the migratory and invasive competence of the cells (Fig. 2D, *P* < 0.01 compared with control). Subsequently, we analyzed MMP12 expression in cervical tumors. Analyses of IHC staining (*P* < 0.01, Fig. 2E), TCGA database (*P* < 0.01, Fig. 2F), and GEPIA database (*P* < 0.05, Fig. 2G) revealed that MMP12 was highly expressed in cervical tumors compared with normal tissues. Moreover, MMP12 expression in cervical tumors was strongly correlated with the decrease in overall survival rate (*P* = 0.036, Fig. 2H). Interestingly, MMP12 expression was significantly correlated with MTA2 expression in cervical tumors (*P* = 0.001, Fig. 2I). We demonstrated the in vivo effects of MMP12-knockdown on the metastasis of cervical cancer cells by using a xenograft mouse model. Results showed that MMP12-knockdown clearly reduced the metastasis and lung weight of SiHa cells to the lungs compared with the shLuc group (Fig. 2J–L). Furthermore, IHC staining suggested that the proliferation marker Ki-67 was considerably weaker in the MMP12 knockdown group (Fig. 2M).

### p38 MAPK is involved in MMP12 downregulation in MTA2-knockdown cervical cancer cells

**Fig. 3:**
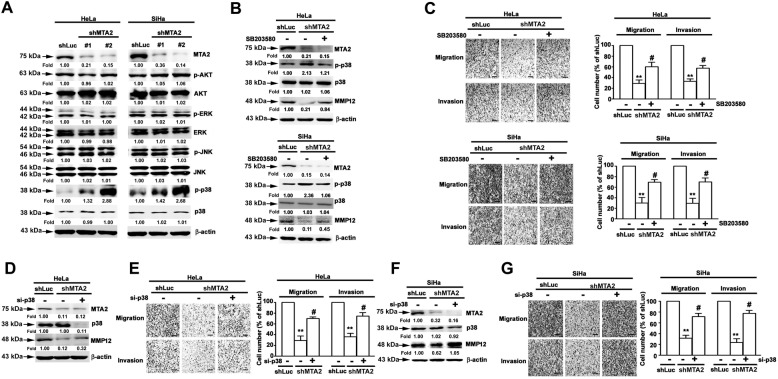
Involvement of p38 signaling in the downregulation of MMP12 in MTA2-knockdown cervical cancer cells. **(A)** Cells were transfected with shRNA against MTA2 (shMTA2) and then lysed for immunodetection of AKT and MAPK activation. (**B**, **C)** Cells were transfected with shMTA2, treated with the p38 inhibitor SB203580, and then subjected to (**B**) immunodetection of p38 activation and MMP12 expression or (**C**) migration and invasion assay. (**D**–**G)** Cells were transfected with shMTA2 and siRNA against p38 (si-p38) and then subjected to (**D**, **F**) immunodetection of MTA2, p38, and MMP12 and (**E**, **G**) migration and invasion assay. β-actin signal was used as internal control. ** and #, *P* < 0.01 and *P* < 0.05 compared with shLuc and shMTA2 cells alone, respectively.

AKT and MAPKs play important role in the regulation of MMPs in different cell types^12,13^. Therefore, we further explored the involvement of AKT and MAPKs in MMP12 downregulation in response to MTA2-knockdown. We assessed the phosphorylation of AKT and three major MAPKs, namely, ERK, JNK, and p38 MAPK (p38), in MTA2-knockdown HeLa and SiHa cells. Only p38 phosphorylation was notably increased, and the protein expression of all the tested kinases did not evidently change (Fig. 3A). We then explored whether p38 activation is involved in MMP12 downregulation and the attenuation of metastatic competence in response to MTA2 knockdown. As shown in Fig. 3B, C, pretreatment with the p38 inhibitor SB203580 clearly inhibited p38 activation and restored MMP12 expression and the cell migration and invasion of MTA2-knockdown HeLa and SiHa cells. Similarly, p38 silencing by specific siRNA downregulated p38 expression and restored MMP12 expression and the metastatic competence of MTA2-knockdown HeLa (Fig. 3D, E) and SiHa (Fig. 3F, G) cells.

### ASK1/MEK3 cascade is involved in p38-downregulated MMP12 expression in MTA2-knockdown cervical cancer cells

**Fig. 4:**
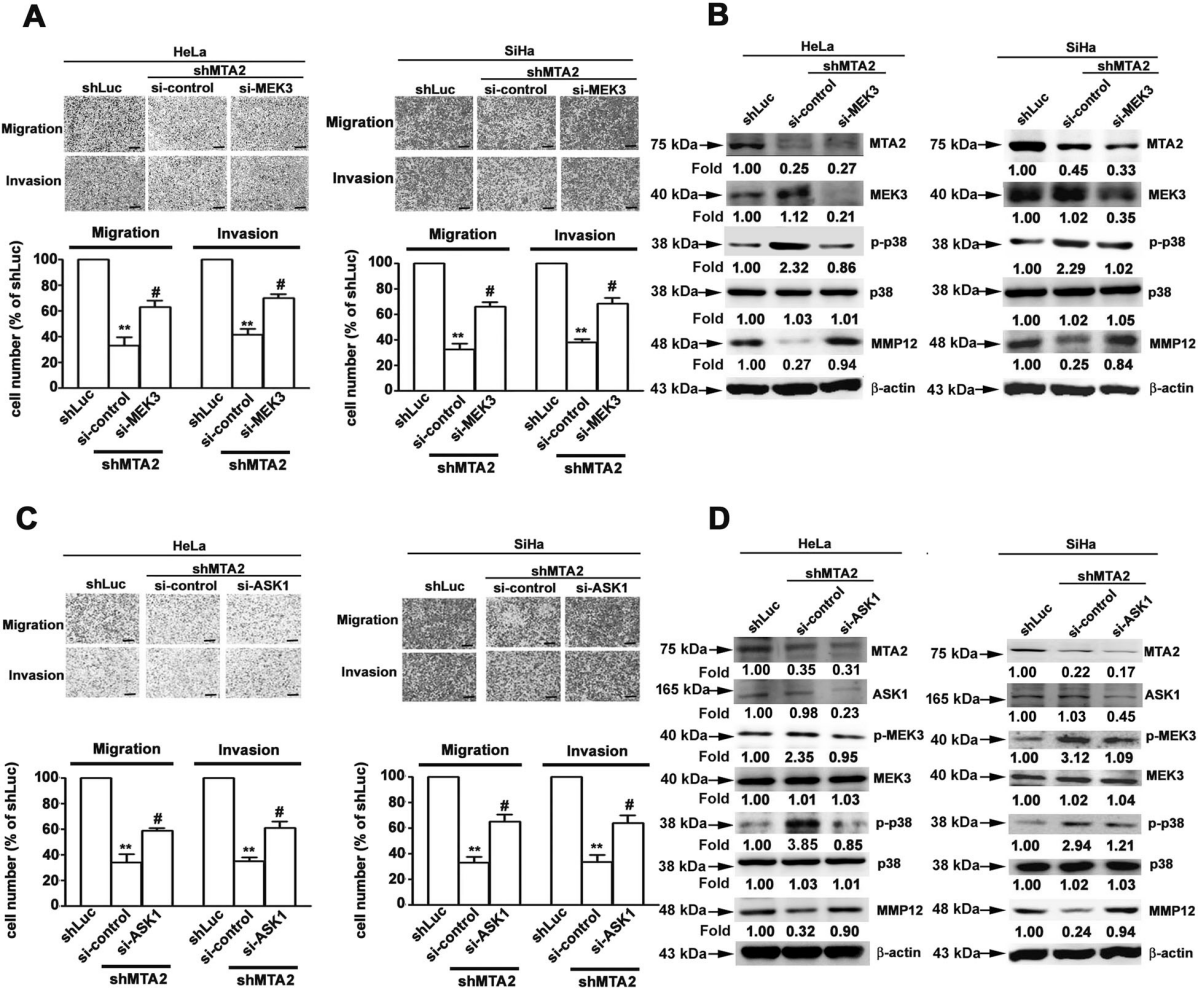
Involvement of ASK1 and MEK3 in p38-governed downregulation of MMP12 in MTA2-knockdown cervical cancer cells. **(A**, **B)** Cells were transfected with shRNA against MTA2 (shMTA2) and siRNA against MEK3 (si-MEK3) and then subjected to (**A**) migration and invasion assay or (**B**) immunodetection of MTA2, MEK3, p38, and MMP12. (**C**, **D)** Cells were transfected with shRNA against MTA2 (shMTA2) and siRNA against ASK1 (si-ASK1) and then subjected to (**C**) migration and invasion assay or (**D**) immunodetection of MTA2, ASK1, MEK3, p38, and MMP12. β-actin signal was used as internal control. ** and #, *P* < 0.01 and *P* < 0.05 compared with shLuc and shMTA2 cells alone, respectively.

On the basis of the observation that MMP12 downregulation is mediated by p38 signaling, we investigated the involvement of the p38 upstream activators MEK3 and ASK1^14^, in MMP12 downregulation and the attenuation of metastatic competence due to MTA2-knockdown. As shown in Fig. 4A, MEK3 silencing by siRNA clearly restored the migratory and invasive potentials of MTA2-knockdown HeLa and SiHa cells (*P* < 0.05 compared with MTA2-knockdown alone). MEK3 silencing also abolished p38 activation and restored MMP12 downregulation in HeLa and SiHa cells in response to MTA2 knockdown (Fig. 4B). Moreover, ASK1 silencing not only restored the metastatic potential of MTA2-knockdown cervical cancer cells (Fig. 4C) and MMP12 expression (Fig. 4D) but also diminished the activation of MEK3 and p38 in response to MTA2 knockdown (Fig. 4D).

### MTA2 knockdown reduces AP1 expression and interferes with AP1 binding to the MMP12 promoter regions (−1801/−1793) in cervical cancer cells

**Fig. 5:**
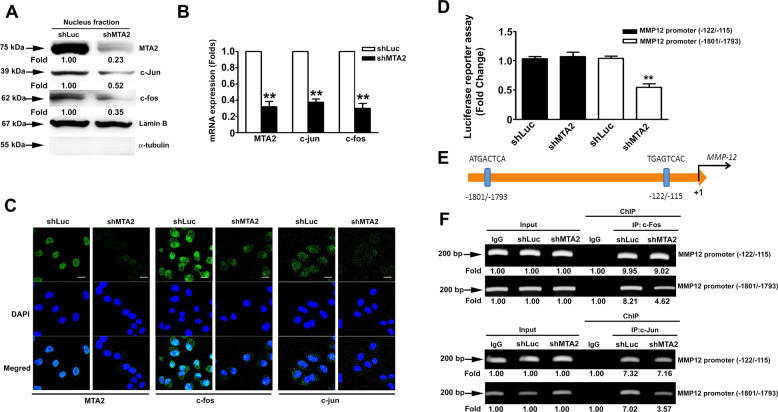
Involvement of AP1(c-Fos/c-Jun) in the downregulation of MMP12 in MTA2-knockdown cervical cancer cells. Cells were infected with shRNA against MTA2 (shMTA2) and then subjected to (**A**) nucleus fractionation and immunodetection of nuclear MTA2 and AP1(c-Fos/c-Jun), (**B**) mRNA expression assessment via qRT-PCR, or (**C**) immunofluorescent detection of MTA2, c-Fos, and c-Jun by confocal microscopy. (**D)** Cells were transfected with luciferase reporter vectors containing the MMP12 promoters (−122/−155) or (−1801/−1793), transfected with shMTA2, and then subjected to report assay. (**E)** Predicted AP1 binding sites on the MMP12 promoter. (**F)** Cells were transfected with shMTA2 and then subjected to ChIP by using antibodies against c-Fos or c-Jun and PCR analysis. **P < 0.01 compared with shLuc cells.

The transcription factor AP1, which consists of c-Jun and c-Fos, plays an important role in controlling MMP1, MMP9, and MMP12 expression in tumor cells^12^. Accordingly, we explored whether AP1 is involved in MMP12 downregulation due to MTA2 knockdown. The AP1 (c-Fos/c-Jun) in nuclear fraction and the mRNA expression of AP1 (c-Fos/c-Jun) in MTA2 knockdown SiHa cells were substantially decreased (Fig. 5A–C). To confirm whether AP1 binds to the predicted binding sites of the MMP12 promoter to exert its transcriptional activity, we constructed two luciferase-expressing vectors containing the sequences of the (−1801/−1793) and (−122/−155) regions of the MMP12 promoter and then transfected the two vectors into MTA2-knockdown SiHa cells. The luciferase activity of the vector with the MMP12 promoter region (−1801/−1793) was clearly reduced in the MTA2-knockdown cells (*P* < 0.01 compared with the control, Fig. 5D). Interestingly, the luciferase activity of the vector with the MMP12 promoter region (−122/−115) was not markedly influenced in the MTA2-knockdown cells (*P* = 0.56 compared with the control, Fig. 5D). To determine which transcription factors regulate and bind to the MMP12 promoter and lead to tumor metastasis, we used the PROMO database (http://alggen.lsi.upc.es) to identify the putative transcription factor binding sites (TFBS) in MMP12 promoter sequences. We predicted that some transcription factors, such as Elk-1, RelA/p65, ERα, and AP-1 (c-Jun and c-Fos), bind to the MMP12 promoter (Supplemental Fig. S2A). Afterward, we analyzed the expression of the transcription factors in shLuc- or shMTA2-SiHa cells via Western blot. Results showed that MTA2 knockdown inhibited the nucleus fraction of c-Jun and c-Fos expression but did not affect the expression of other transcription factors (Elk-1, RelA/p65, and ERα) (Supplemental Fig. S2B). We further analyzed two predicted AP1 binding sites at the (−1801/−1793) and (−122/−155) regions in the MMP12 promoter (Fig. 5E). Chromatin immunoprecipitation assay also revealed that the binding of AP1(c-Fos/c-Jun) to the MMP12 promoter region (−1801/−1793) was clearly reduced in MTA2-knockdown cells (Fig. 5F). By contrast, the binding of AP1(c-Fos/c-Jun) to the MMP12 promoter region (−122/−115) was not affected (Fig. 5F). According to these results, AP-1 plays an important role in MTA2-mediated MMP12 expression in cervical cancer cells.

### MTA2 knockdown attenuates AP1-governed MMP12 expression and inhibits the metastatic potential of cervical cancer cells attributed to ASK1/MEK3/p38-mediated YB1 phosphorylation

**Fig. 6:**
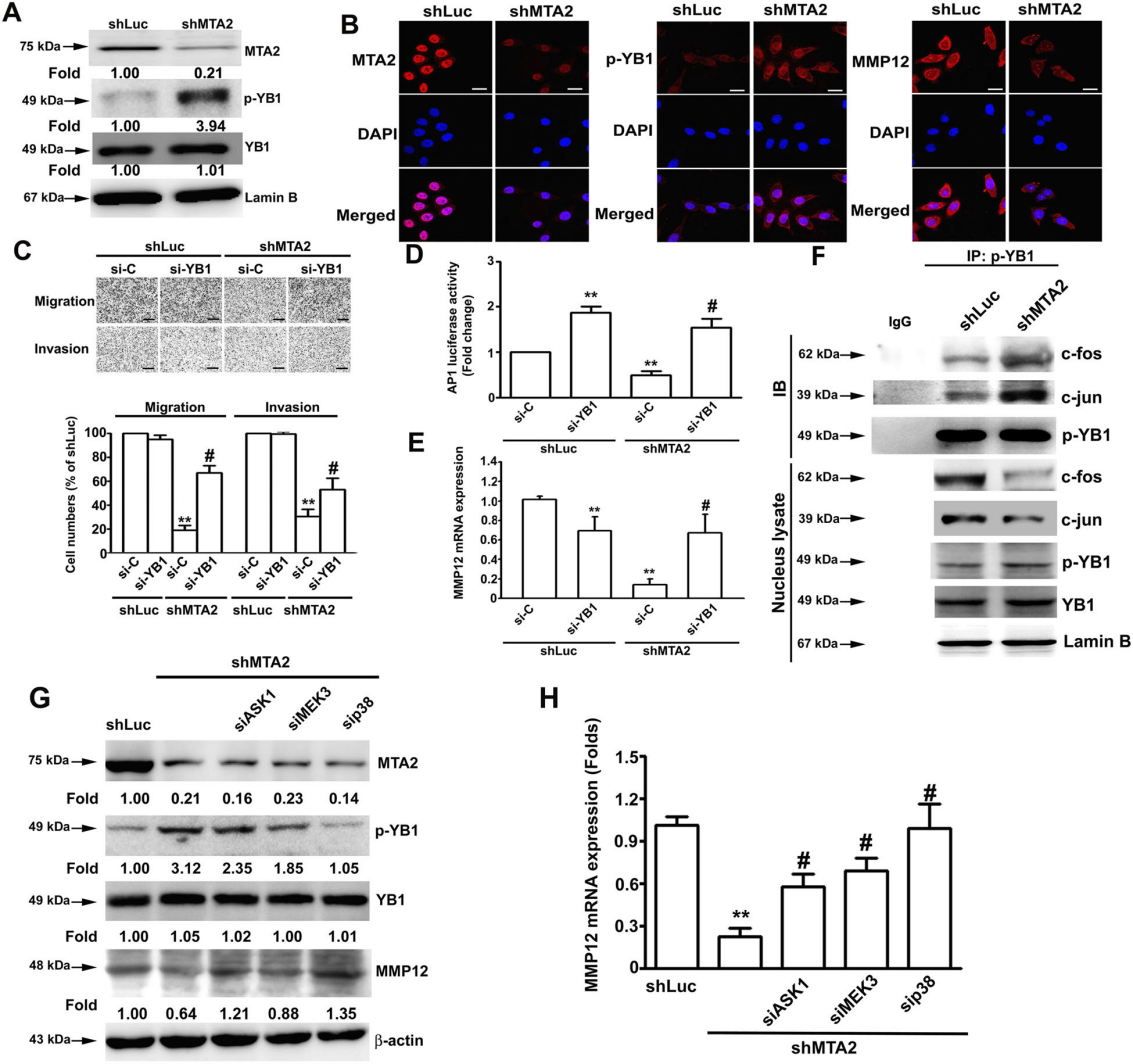
Involvement of YB1 phosphorylation in MMP12 downregulation and attenuation of metastatic potentials in MTA2-knockdown SiHa cells. SiHa cells were transfected with shRNA against MTA2 (shMTA2) and then subjected to (**A**) nucleus fractionation and immunodetection of nuclear MTA2, phosphylated YB1 (p-YB1), and YB1 or (**B**) immunofluorescent detection of MTA2, p-YB1, and MMP12 by confocal microscopy. (**C**–**E)** Cells were transfected with shMTA2 and siRNA against YB1 (si-YB1) and then subjected to (**C**) migration and invasion assay, (**D**) AP1 reporter assay, or (**E**) MMP12 mRNA assessment via qRT-PCR. **F** Cells were transfected with shMTA2 and then subjected to immunoprecipitation by using anti-p-YB1 antibody and immunodetection of the indicated poteins. (**G**, **H)** Cells were transfected with shMTA2 combined with siRNA against ASK1 (si-ASK1), MEK3 (si-MEK3), or p38 (si-p38) and then subjected to (**G**) immunodetection of the indicated proteins or (**H**) MMP12 mRNA expression assessment via qRT-PCR. ** and #, *P* < 0.01 and *P* < 0.05 compared with shLuc and shMTA2 cells alone, respectively.

YB1 reportedly interacts with the AP1-binding site on DNA and represses AP1-dependent MMP12 expression^15^. We explored the role of YB1 in the suppressed migration and invasion of cervical cancer cells in response to MTA2 knockdown. As shown in Fig. 6A, B, MTA2 knockdown remarkably induced the phosphorylation of p-YB1 and promoted the nuclear translocation of p-YB1 in SiHa cells compared with the control. Furthermore, MTA2/YB1 double knockdown restored the migratory and invasive potentials of SiHa cells compared with MTA2 knockdown alone (Fig. 6C, *P* < 0.05). Moreover, MTA2/YB1 double knockdown clearly restored the diminished AP1 promoter activity and the mRNA expression of MMP12 in SiHa cells compared with MTA2 knockdown alone (Fig. 6D, E, *P* < 0.05). The interaction between p-YB1 and AP1(c-fos/c-jun) was also demonstrated via immunoprecipitation assay. Results showed that MTA2 knockdown considerably increased p-YB1 levels and decreased AP1(c-fos/c-jun) levels in the nuclear fraction of SiHa cells (Fig. 6F). Finally, we examined the involvement of ASK1, MEK3, and p38 in the phosphorylation of YB1 and the inhibition of MMP12 expression in response to MTA2 knockdown. Results showed that silencing of ASK1, MEK3, or p38 clearly reduced YB1 phosphorylation (Fig. 6G) and restored MMP12 expression (Fig. 6H, *P* < 0.05) in the MTA2-knockdown SiHa cells.

## Discussion

Metastasis is the major cause of death in patients with cancer. It is the key cellular process that disseminates cancer cells from the primary tumor to distant areas. MTA2 is overexpressed in several solid tumors, such as gastric cancer and papillary thyroid cancer, and is associated with their metastases^16,17^. Our previous study also revealed that MTA2 is overexpressed in hepatocellular carcinoma (HCC), and MTA2 knockdown reduces the mestastasis of HCC cell lines by inhibiting MMP2 expression^18^. In the present study, we further demonstrated that silencing MTA2 clearly inhibits the metastasis of cervical cancer cells by inducing the ASK1/MEK3/p38/YB-1 axis and the consequent suppression of MMP12. We also showed that MTA2 is highly expressed in cervical tumors, suggesting that MTA2/MMP12 could be a potential poor prognostic marker for cervical cancer.

ASK1 is an ubiquitously expressed MAP kinase that can activate the JNK and p38 signaling pathways and induce cell apoptosis in response to various extracellular stresses, such as oxidative stress and cytokine-induced apoptosis^14,19,20^. Here, we observed that ASK1 activation is crucial to the downregulation of MMP12 through the MEK3/p38 cascade in response to MTA2 knockdown, suggesting that the inhibited ASK1/MEK3/p38 axis may play an important role in the progression of cervical cancer.

Y-box binding protein 1 (YB1) is a multifunctional protein that controls the transcription and translation of various genes and proteins involved in several important cell physiological activities, including proliferation^21^, survival^22,23^, DNA replication^24^ and repair^25^, multidrug resistance^26^, and epithelial to mesenchymal transition^27^. Nuclear translocation of YB1 is induced by the phosphorylation at serine-102^28^, and the nuclear-phosphorylated YB1 exerts its transcription regulatory activity to regulate the expression of proliferative genes, including EGFR^29^, PCNA^30^, and Cyclin A/B^31^. The purported roles of YB1 in carcinogenesis are controversial, and this protein reportedly has oncogenic- and tumor-suppressing activities^16,32^. Here, our findings revealed that MTA2 knockdown induces p38-mediated YB1 phosphorylation at serine-102 and the subsequent nuclear translocation of phosphorylated YB1. The phosphorylated YB1 then interacts with AP1 to disrupt the transcriptional activity of AP1, leading to the downregulation of MMP12 in cervical cancer cells. These findings suggested that YB1 may have tumor-suppressing activity by disrupting AP1-governed MMP12 expression in cervical cancer cells.

Upregulation of MMP2 and MMP9 is highly associated with tumor progression; thus, both of them are regarded as potential targets for cancer therapy^33^. MMP12 overexpression is also associated with the recurrence and metastasis in non-small cell lung cancer^32^ and glioma^34^. In addition, MMP12 is overexpressed in HPV-positive cervical cancer cell lines, suggesting that it is involved in the pathogenesis of cervical cancer^35^. Similarly, we observed that the higher expression of MMP12 is linked to lower survival probability of patients with cervical cancer. Moreover, we demonstrated that MTA2 knockdown can downregulate MMP12 expression and the metastatic potential of HPV-positive cervical cancer cells. Collectively, these findings indicated that MTA2 plays a key role in regulating MMP12 expression in HPV-positive cervical cancer cells, suggesting that MTA2 may be involved in the HPV-induced carcinogenesis of cervical cancer.

**Fig. 7:**
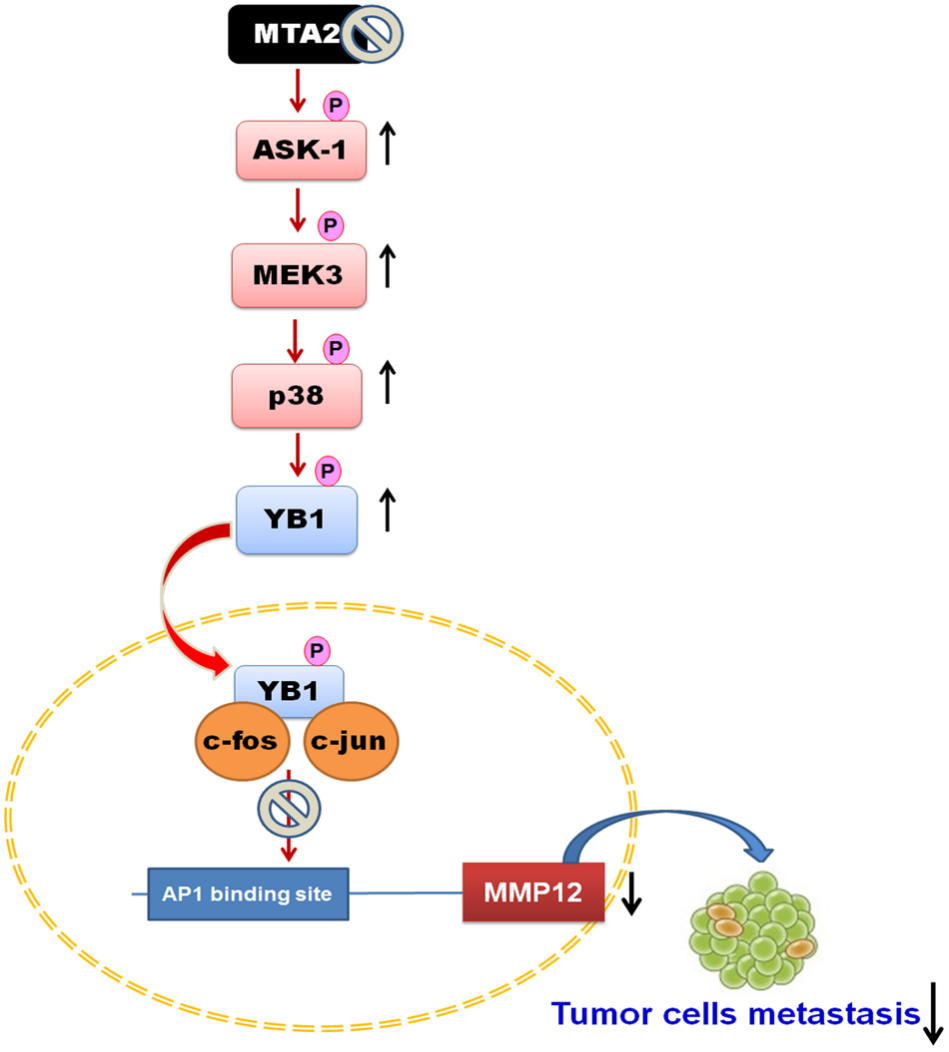
Proposed mechanism for the MTA2 knockdown attenuating metastatic potential of cervical cancer cell. Our findings indicate that MTA2 knockdown induces activation of the ASK1/MEK3/p38 cascade, which subsequently leads to YB1 phosphorylation and the binding of p-YB1 and AP1, inhibiting AP1 transcriptional activity and MMP12 expression, thereby reducing cervical cancer metastasis.

In conclusion, this study revealed that MTA2 and MMP12 are highly expressed in cervical carcinomas, and knockdown of MTA2 attenuates the metastatic ability of cervical cancer cells, both of which are attributed to the activation of the ASK1/MEK3/p38/YB1 axis and the inhibition of AP1-mediated MMP12 expression (Fig. 7). These findings not only highlight the role of MTA2 in the metastasis of cervical cancer but also suggest that MTA2 may be a potential target for the treatment of cervical cancer.

## Supplementary information

Supplementary Figure S1–S2
