# Supplementary material for: MTA2 silencing attenuates the metastatic potential of cervical cancer cells by inhibiting AP1-mediated MMP12 expression via the ASK1/MEK3/p38/YB1 axis
Source: Cell Death Dis. 2021 May 6;12(5):451. doi: 10.1038/s41419-021-03729-1 (PMC8102478; doi:10.1038/s41419-021-03729-1)
Supplement: Supplementary file 1 — Supplementary Figure S1–S2 [file 41419_2021_3729_MOESM1_ESM.docx]

**MTA2 silencing attenuates the metastatic potential of cervical cancer cells by inhibiting AP1-mediated MMP12 expression via the ASK1/MEK3/p38/YB1 axis**

Chia-Liang Lin , Tsung-Ho Ying, Shun-Fa Yang, Hui-Ling Chiou, Yong-Syuan Chen, Shao-Hsuan Kao, Yi-Hsien Hsieh

**Supplementary Figure 1**


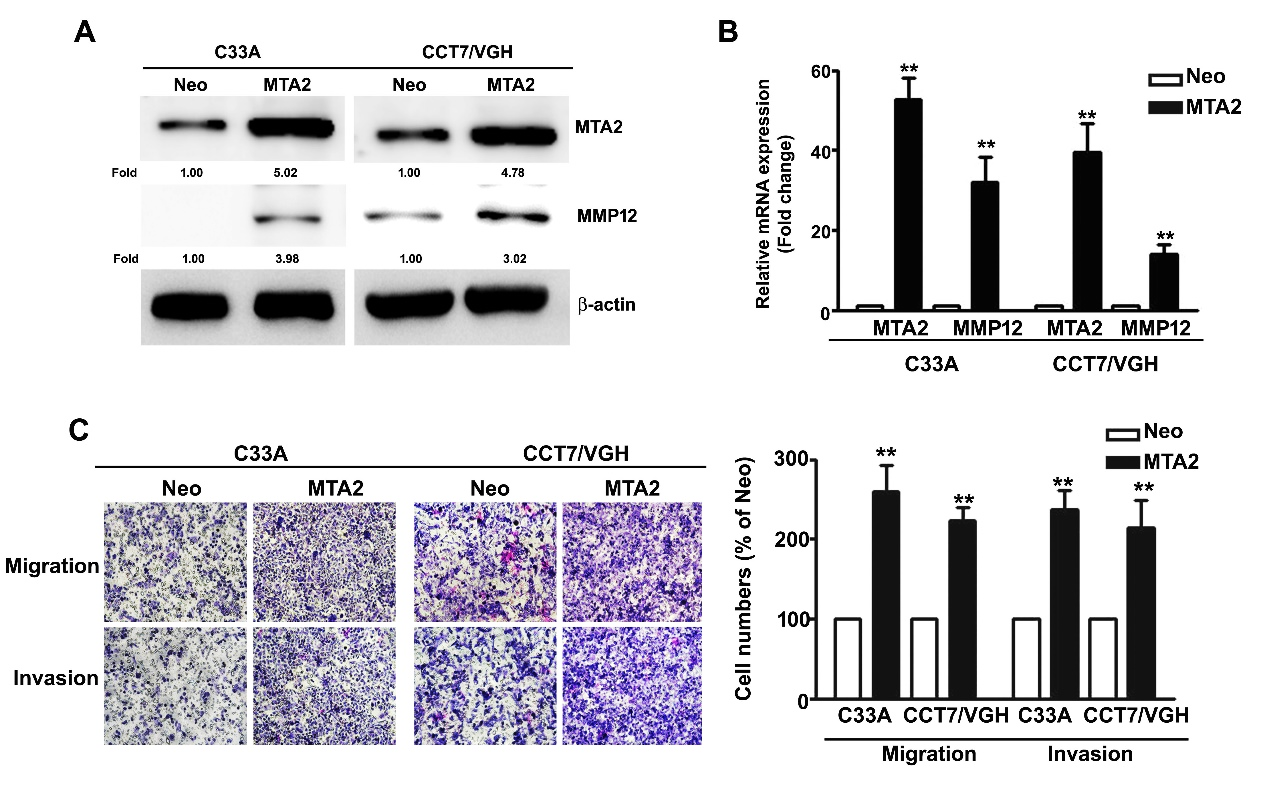


**Supplementary Figure S1**: Overexpression of MTA2 promotes the migration and invasion of human cervical cancer cells. C33A or CCT7/VGH cells were transfected

with Neo or MTA2 plasmid, respectively. (A) The protein expression of MTA2 and MMP12 were detected by immunoblotting. (B) qRT-PCR assay to detect the mRNA expression of MTA2 and MMP12. (C) Migratory and invasive potential of C33A or CCT7/VGH cells. Data were presented as the mean ± SE of at least three independent experiments. **, represented p < 0.01 comparing to that of Neo cells.

**Supplementary Figure 2**


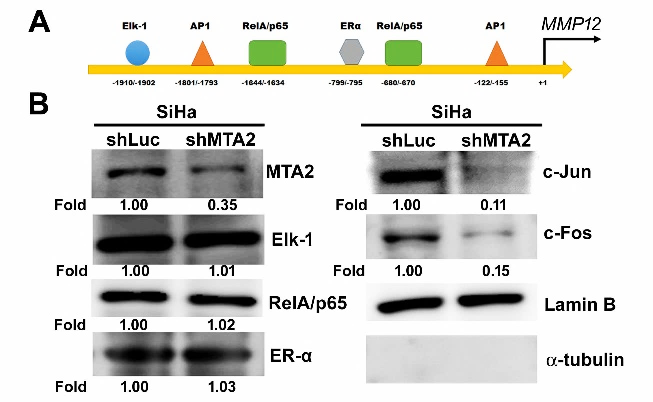


**Supplementary Figure S2**: Inhibition of c-Jun and c-Fos transcription factor expression in Knockdown MTA2-SiHa cells. (A) PROMO database to identify of putative transcription factor binding sites (TFBS) in MMP12 promoter sequences. (B) Cells were infected with shRNA against Luc (shLuc) or MTA2 (shMTA2), and then subject to nucleus fractionation and immunoblotting of nuclear MTA2, Elk-1, Rel/p65, ER-α, and AP1(c-Fos/c-Jun) expression. Lamin B as nucleus marker; α-tubulin as cytoplasmic marker.
